# Comparative Analysis of AI Models in Predicting Treatment Strategies for Unruptured Intracranial Aneurysms

**DOI:** 10.3390/brainsci15101061

**Published:** 2025-09-29

**Authors:** Manou Overstijns, Sameer Nazeeruddin, Pierre Scheffler, Roland Roelz, Jürgen Beck, Amir El Rahal

**Affiliations:** 1 Department of Neurosurgery, Faculty of Medicine, Medical Centre, University of Freiburg, Breisacher-Str. 64, 79106 Freiburg, Germany; manou.overstijns@uniklinik-freiburg.de (M.O.); sameer.nazeeruddin@uniklinik-freiburg.de (S.N.); pierre.scheffler@uniklinik-freiburg.de (P.S.); roland.roelz@uniklinik-freiburg.de (R.R.); amir.elrahal@uniklinik-freiburg.de (A.E.R.); 2 Faculty of Medicine of Geneva, University of Geneva, 1201 Geneva, Switzerland

**Keywords:** artificial intelligence, unruptured intracranial aneurysm, treatment prediction, AI model comparison, predictive accuracy

## Abstract

**Objectives:** The increasing incidence of unruptured intracranial aneurysms (UIAs) has led to significant demands on neurovascular boards. Large language models (LLMs), such as ChatGPT-4, ChatGPT-3.5, Claude, and Atlas GPT, have emerged as tools to support clinical decision-making. This study compares treatment recommendations from these AI models with those of an interdisciplinary neurovascular board to evaluate their accuracy and alignment. **Methods:** We retrospectively included all 57 patients with UIAs discussed by the neurovascular board in 2023. The board’s consensus decision served as the reference standard. Key clinical and radiographic data, including PHASES, ELAPSS, and UIATS scores, were provided to the AI models. Each model was tasked with recommending either conservative or operative management and specifying the treatment modality (clipping, coiling, flow diverter, or WEB device/flow diverter) where appropriate. AI model recommendations were compared with the board’s decisions for management and the specific treatment modality of the UIA. **Results:** ChatGPT-4 achieved the highest accuracy in correctly predicting conservative or operative management (89%) and specific treatment types (73%), followed by Atlas GPT (74% accuracy in conservative/operative decisions and 55% accuracy in specific treatment types), Claude (70% accuracy in conservative/operative decisions and 50% accuracy in specific treatment types), and ChatGPT-3.5 (82% accuracy in conservative/operative decisions and 27% accuracy in specific treatment types). ChatGPT-3.5 displayed a strong preference for clipping (94.3%). ELAPSS scores significantly influenced AI recommendations and decision-making, particularly for ChatGPT-4 and ChatGPT-3.5. Follow-up recommendations for conservative management were shorter among AI models, with Claude suggesting the shortest interval (7.72 months) compared to the neurovascular board’s 13.36 months. **Conclusions:** AI models, particularly ChatGPT-4, align closely with expert neurovascular board decisions and offer promising support for initial clinical decision-making, particularly in resource-limited settings. However, interdisciplinary neurovascular boards remain unreplaceable for UIA management, and AI should be viewed as a complementary tool. The observed improvement from ChatGPT-3.5 to ChatGPT-4 underscores the rapid evolution of AI technology, and further advancements are expected to enhance both performance and accuracy in the future.

## 1. Introduction

Unruptured intracranial aneurysms (UIAs) affect approximately 3% of the general population, often remaining asymptomatic and undetected until discovered incidentally through neuroimaging performed for unrelated reasons [1,2]. The increasing accessibility of advanced imaging techniques has led to a higher incidence of incidental UIA detection, posing significant challenges in clinical decision-making regarding their management [3]. The primary dilemma revolves around whether to intervene or manage the aneurysm conservatively, a decision that necessitates a careful balance between the risk of aneurysm rupture and the potential complications associated with treatment [4,5,6].

Management strategies for UIAs are inherently complex and require close interdisciplinary collaboration between neurosurgeons, neuroradiologists, and neurologists. A range of patient-specific factors, including age, comorbidities, family history, and aneurysm characteristics such as size, location, morphology, and documented growth patterns, guide decisions [4,7]. Treatment options encompass conservative treatment, surgical clipping, endovascular coiling, flow diversion, and the use of WEB (Woven EndoBridge) devices, each with its risk–benefit profile [8,9,10].

To aid in the decision-making process, predictive scoring systems have been developed. The PHASES score estimates the 5-year risk of aneurysm rupture based on population, hypertension, age, aneurysm size, a history of earlier subarachnoid hemorrhage from another aneurysm, and aneurysm site [11]. The ELAPSS score predicts the risk of aneurysm growth based on factors such as patient age, aneurysm size, and location [12]. The Unruptured Intracranial Aneurysm Treatment Score (UIATS) integrates both patient- and aneurysm-related factors to provide a comprehensive assessment for recommending either intervention or observation [13].

Even with established decision-making aids, the rising number of incidental UIA detections can place a considerable burden on neurovascular teams, sometimes leading to resource constraints and delayed specialist consultations [14]. In many clinical settings, immediate access to an interdisciplinary neurovascular board may not be readily available, highlighting the need for supplementary forms of preliminary decision support. In this context, artificial intelligence (AI), particularly Large Language Models (LLMs), has emerged as a promising adjunct. Models such as ChatGPT-4 (OpenAI), ChatGPT-3.5. Claude (Anthropic) and Atlas GPT (Atlas Meditech) utilize deep learning algorithms and extensive medical literature to generate human-like language outputs that can aid in guiding initial clinical decisions [15].

Recent studies have explored the application of AI and LLMs in neurosurgical decision-making, particularly in neuro-oncology. For instance, investigations into ChatGPT’s ability to provide diagnoses and treatment plans for brain tumors have demonstrated its potential to assist in clinical evaluation [16]. However, the use of LLMs in determining management strategies for UIAs has not been extensively studied. This study aims to compare the decisions made by an interdisciplinary neurovascular board with those generated by four different LLMs—ChatGPT-4, ChatGPT-3.5, Claude, and Atlas GPT—regarding the optimal management of unruptured intracranial aneurysms.

## 2. Methods

### 2.1. Study Design

Ethical approval was obtained from the local ethics committee (protocol code EK-Freiburg: 24-1429-S1, approval date 12 November 2024). The requirement for individual informed consent was waived because the study involved a retrospective analysis of pseudonymized patient data, which did not allow direct identification of individuals. We adhered to the STROBE (Strengthening the Reporting of Observational Studies in Epidemiology) checklist guidelines to ensure comprehensive and transparent reporting [17]. All data is available on reasonable request to the authors.

Patients were included if (i) UIA was confirmed using neuroimaging (either digital subtraction angiography, computed tomography angiography, or magnetic resonance angiography), (ii) they were discussed in our interdisciplinary neurovascular board between 1 January 2023, and 31 December 2023, and (iii) were aged 18 years or above. Patients were excluded if (i) other vascular pathologies were present (e.g., arteriovenous malformation), (ii) patients had ruptured aneurysms, and iii) patients without definitive management decisions from the board (3 patients in total). Excluding these cases ensured that all included patients had clear expert decisions, which was essential for accurate comparison with the Large Language Models (LLMs).

### 2.2. Expert Decision

All cases were reviewed in our institutional interdisciplinary neurovascular board, which is convened on a weekly basis. For each case, at least one senior specialist from each core discipline was present: a neuroradiologist, a neurointerventionalist, a neurologist, and a neurosurgeon. The estimated 5-year aneurysm rupture risk, primarily assessed using the PHASES score, was weighed against the estimated treatment risk, taking into account patient age, comorbidities (with special consideration of severe comorbidities defined as ASA ≥ 3), aneurysm morphology (saccular or fusiform), size, location, irregularity, and documented growth [11]. In cases where rupture risk exceeded expected procedural risk, interventional treatment was favored. Additional factors included the ELAPSS score to estimate growth risk, anatomical considerations for surgical versus endovascular feasibility, and any history of previous subarachnoid hemorrhage. The final decision reflected consensus among all present board members, integrating quantitative risk scores with qualitative expert judgment in accordance with current international guidelines [4,18].

### 2.3. Data Collection

**Figure 1:**
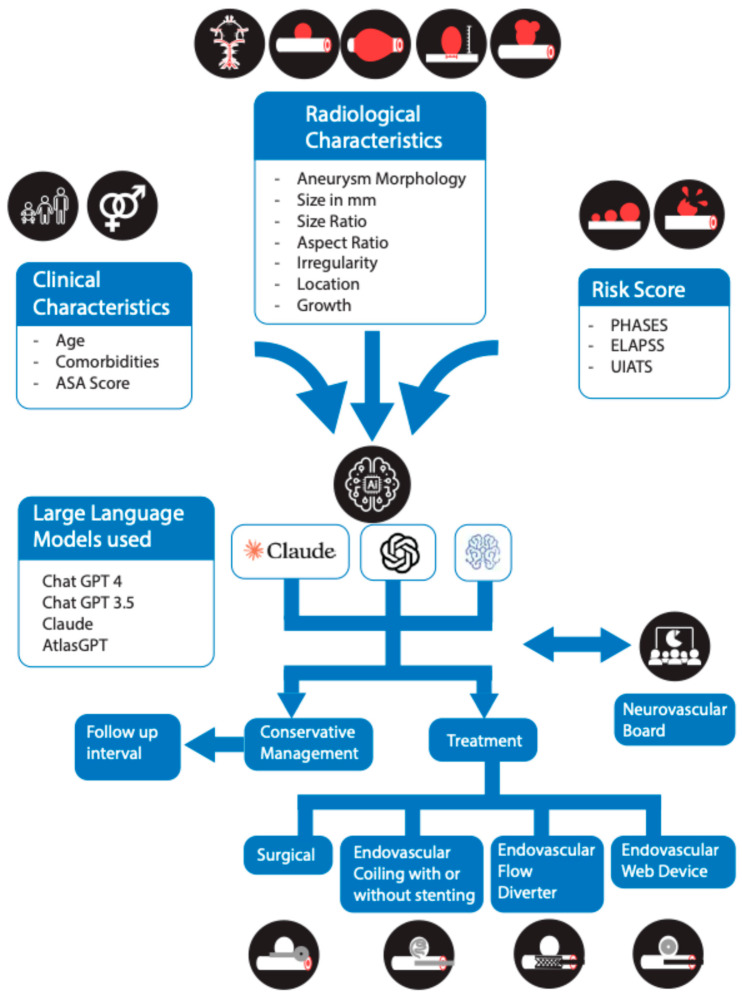
Flowchart depicting clinical, radiological, and risk-score data fed into Large Language Models (GPT-4, GPT-3.5, Claude, Atlas GPT) to generate recommendations for UIA management. The models propose either conservative follow-up or interventional treatment (surgical or endovascular), which is then compared against the expert neurovascular board’s decisions.

We collected key patient characteristics essential for determining the management of unruptured intracranial aneurysms (Figure 1). These data were subsequently entered into four Large Language Models (LLMs) to obtain management recommendations: ChatGPT-4 (OpenAI, GPT-4.0), ChatGPT-3.5 (OpenAI, GPT-3.5-turbo), Claude (Anthropic, Claude 2.1), and Atlas GPT (Atlas Meditech, Atlas GPT v1.3). All models were accessed and queried in October 2024, ensuring that each was evaluated in the same time frame. Specifying the exact model versions and access date is essential for reproducibility, as LLM performance and outputs can change substantially with updates. The collected data included patient age, presence of severe comorbidities, aneurysm morphology (saccular or fusiform), and aneurysm irregularity. We also recorded whether the aneurysm was intradural or extradural, its maximum size in millimeters, anatomical location, presence of multiple aneurysms, size ratio, aspect ratio, and whether an aneurysmal growth was identified. Additionally, we calculated key risk assessment scores for each patient, including PHASES, ELAPSS, UIATS, and American Society of Anesthesiology (ASA) score, to create a comprehensive profile that guides management decision [11,12,13].

To ensure consistency, we prompted each Large Language Model (LLM), ChatGPT-4, ChatGPT-3.5, Claude, and Atlas GPT, with a standardized request (Supplementary data S1). We instructed the models to analyze each patient’s clinical and radiographic data and to recommend either conservative management or treatment for unruptured intracranial aneurysms. When recommending treatment, the models were asked to specify the modality (surgical or endovascular) and, if endovascular, to further detail the specific technique (coiling with or without stenting, flow diverter, or web device). For conservative management recommendations, we asked the models to indicate a suggested follow-up time.

### 2.4. Data Input and Prompt Design

To ensure consistency and minimize variability in the AI-generated recommendations, we developed a standardized input format that was uniformly presented to each Large Language Model (LLM). This standardized format included key patient and aneurysm characteristics, as well as relevant risk scores (e.g., PHASES, ELAPSS), mirroring the clinical data used in neurovascular board discussions. By providing an identical dataset and prompt wording to each model, we aimed to obtain responses that could be directly compared with one another and with the decisions of our neurovascular board.

Because LLMs like ChatGPT-4 and ChatGPT-3.5, as well as other models, are heavily influenced by the nature and detail of the input provided, adopting a methodical and structured input design was critical for generating reproducible outputs. Each case was presented in tabular form, describing patient demographics, aneurysm morphology and metrics, as well as the scoring systems for rupture risk and growth risk. Each model was queried with a standardized prompt that explicitly asked for a single preferred management decision. Outputs were subsequently mapped into five predefined categories: conservative management, surgical clipping, endovascular coiling, flow diverter, or WEB device. If a model proposed several options but expressed a preference (for example, “clipping would be preferred, though coiling could also be considered”), the preferred option was coded. In cases where hedging language was used, a follow-up clarification was requested, and the final stated preference was recorded. Free-text terminology was standardized to the closest category (for example, “microsurgical occlusion” was classified as clipping, and “endovascular embolization with coils” was classified as coiling). With this structured approach, each model ultimately provided a clear recommendation that could be assigned consistently to one of the predefined treatment categories.

### 2.5. Statistical Analysis

Statistical analysis was performed using SPSS, version 24 (IBM Corp, Armonk, NY, USA). To assess the accuracy of each LLM, we used a binary outcome (1 = correct, 0 = incorrect) to indicate alignment with the neurovascular board’s decision. We then used Cochran’s Q test to evaluate the significance of differences in accuracy across the four models. Subsequently, pairwise comparisons between the LLMs were performed using the McNemar test. Given the multiple comparisons, we applied a Bonferroni correction to control for Type I error.

## 3. Results

### 3.1. Demographics and Aneurysm Characteristics

A total of 57 patients with unruptured intracranial aneurysms (UIAs) were included in the study after applying the inclusion and exclusion criteria (Table 1). The mean age of the patients was 66.2 years. In total, 53 (93.0%) aneurysms were saccular in morphology, with 3 (5.3%) cases classified as fusiform. Forty-eight aneurysms (84.2%) were located intradurally, while nine cases (15.8%) were extradural [19].

The median maximum aneurysm size was 6.0 mm, ranging from 2.0 to 25.0 mm. In terms of location, aneurysms were found in the middle cerebral artery (MCA) in 16 cases (28.1%), anterior communicating artery (ACOM) in 13 cases (22.8%), internal carotid artery (ICA), including posterior communicating artery, in 15 cases (26.3%), vertebral artery and posterior cerebral artery in 3 cases (5.3%), basilar artery in 7 cases (12.3%), and other locations in 3 cases (5.3%). Thirteen patients (22.8%) had multiple aneurysms, and 4 patients (7.0%) showed documented growth of the aneurysm over time.

The calculated risk scores for each patient provided additional context for management decisions. The mean PHASES score was 6.9, ranging from 0 to 16, while the mean ELAPSS score was 12.1, with a range from 2 to 29. The Unruptured Intracranial Aneurysm Treatment Score (UIATS) favored intervention in 30 patients (52.6%) and conservative management in 27 patients (47.4%).

Expert interdisciplinary consensus recommended conservative treatment for 26 (45.6%) patients, and 31 (54.3%) patients were recommended for treatment of the aneurysm. Among those advised to undergo treatment, 14 patients (24.6%) were recommended for operative clipping, and 13 patients (22.8%) were advised to undergo endovascular coiling. Additionally, 2 patients (3.5%) received recommendations for treatment with a flow diverter, and another 2 patients (3.5%) were advised to use the WEB device/flow diverter.

ChatGPT-3.5 advocated treatment of the aneurysm the most (61.4%), followed by ChatGPT-4 (57.9%), Claude (52.6%), and Atlas GPT (49.1%). Regarding treatment types, ChatGPT-3.5 showed a strong preference for clipping (57.9%), whereas ChatGPT-4 demonstrated a more balanced distribution between clipping (22.8%) and coiling (29.9%). Claude and Atlas GPT leaned toward coiling (33.3% and 29.9%, respectively) and conservative management (42.1% and 50.9%, respectively).

For follow-up recommendations in conservative management, the neurovascular board suggested a mean interval of 13.36 months (SD = 5.84). ChatGPT-3.5 consistently recommended a 12-month follow-up (SD = 0), while GPT-4 and Atlas GPT proposed shorter intervals of 10.00 months (SD = 2.89) and 10.76 months (SD = 2.47), respectively. Claude advised the shortest follow-up period, with a mean of 7.72 months (SD = 3.60) (Table 2).

### 3.2. Accuracy of AI Models in Predicting Conservative Treatment

**Figure 2:**
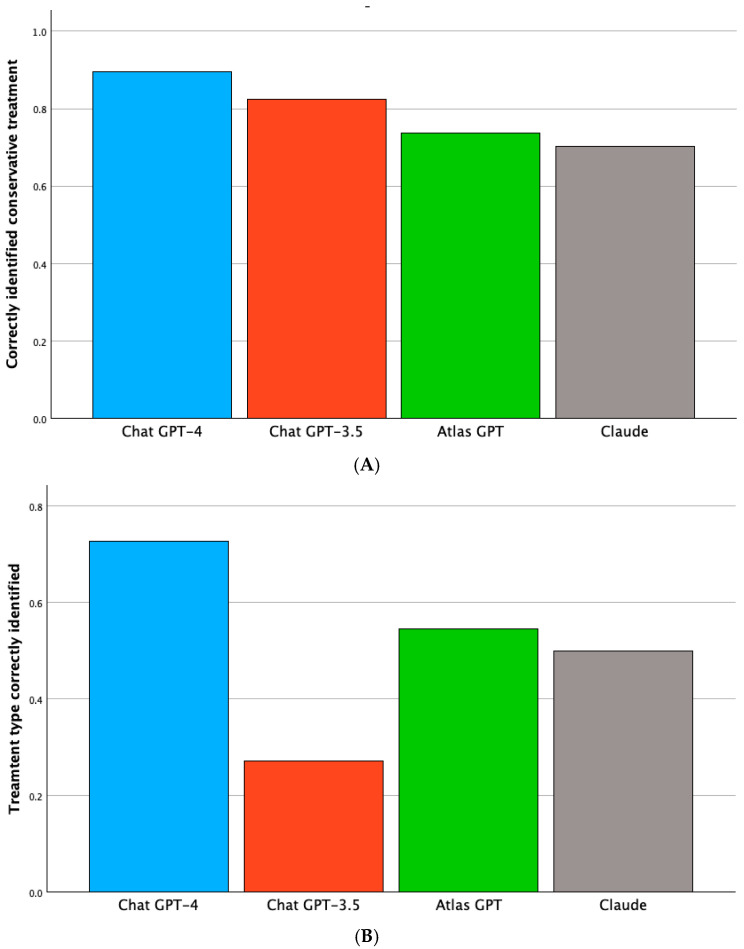
(**A**) Accuracy of each AI model in correctly identifying conservative treatment recommendations for unruptured intracranial aneurysms. ChatGPT-4 achieved the highest accuracy, followed by ChatGPT-3.5, Atlas GPT, and Claude. (**B**) Accuracy of each AI model in correctly identifying the specific treatment type for unruptured intracranial aneurysms.

In predicting the possibility for conservative treatment, ChatGPT-4 had the highest accuracy, correctly aligning with the neurovascular board’s recommendation 89% of the time, followed by ChatGPT-3.5 at 82%, Atlas at 74%, and Claude at 70% (Table 3A and Figure 2A). Cochran’s Q test demonstrated a statistically significant difference among the models (*p* = 0.003). Pairwise comparisons with the Bonferroni adjustment indicated that ChatGPT-4 was significantly more accurate than both Claude (*p* < 0.001) and Atlas (*p* = 0.002). At the same time, the differences between GPT-4 and GPT-3.5, as well as between GPT-3.5 and Claude, were not statistically significant.

The recommendations for follow-up intervals among patients advised to undergo conservative management varied across the neurovascular board and each AI model. The neurovascular board recommended a mean follow-up interval of 13.36 months (SD = 5.84). ChatGPT-4 suggested a slightly shorter follow-up period, with a mean of 10 months (SD = 2.89), while ChatGPT-3.5 consistently recommended a 12-month interval for all cases (resulting in a standard deviation of 0). Claude advised a shorter follow-up interval than the others, with a mean of 7.72 months (SD = 3.60). Atlas GPT’s recommendations were closer to ChatGPT-4, with a mean follow-up interval of 10.76 months (SD = 2.47). Compared with the neurovascular board, this represents significant mean reductions of approximately 3.4 months for ChatGPT-4, 1.4 months for ChatGPT-3.5, 5.6 months for Claude, and 2.6 months for Atlas GPT (all *p* < 0.001).

There was no significant difference in the percentage of conservative treatment recommendations between the neurovascular board and the AI models (ChatGPT-4, ChatGPT-3.5, Claude, and Atlas GPT). Cochran’s Q test showed a result of 5.214 (df = 4, *p* = 0.266). The mean rates of conservative recommendations were 45.6% for the neurovascular board, 42.1% for ChatGPT-4, 38.6% for ChatGPT-3.5, 42.1% for Claude, and 50.9% for Atlas GPT. These results suggest that the AI models were neither more conservative nor more aggressive compared to the neurovascular board.

### 3.3. Accuracy of AI Models in Predicting Specific Treatment Type

The accuracy of each AI model in correctly matching the neurovascular board’s recommendation for the specific treatment modality (Operative clipping, endovascular coiling, flow diverter, or WEB device) is shown in Table 3B and Figure 2B. ChatGPT-4 again achieved the highest accuracy at 73%, followed by Atlas GPT (55%) and Claude (50%). ChatGPT-3.5 had the lowest accuracy at 27%. Cochran’s Q test revealed a significant difference across the models (*p* = 0.008). Pairwise comparisons with Bonferroni adjustment showed that ChatGPT-4 performed significantly better than ChatGPT-3.5 (*p* = 0.002), though differences between ChatGPT-4 and Claude or Atlas GPT were not statistically significant. ChatGPT-4 again showed the highest accuracy, with Atlas GPT and Claude performing moderately well, while ChatGPT-3.5 had the lowest accuracy.

### 3.4. Factors Influencing AI Model Predictions for Treatment

To determine the factors influencing AI model decisions for the treatment of unruptured intracranial aneurysms, a multivariate logistic regression analysis was performed. In the multivariable analysis, the dependent variable was whether the AI model’s treatment recommendation (surgical vs. conservative) matched the neurovascular board’s decision for each patient. In this analysis, the ELAPSS score emerged as a significant predictor of AI model accuracy for both ChatGPT-4 (OR = 1.37, 95% CI: 1.11–1.69, *p* = 0.003) and ChatGPT-3.5 (OR = 1.40, 95% CI: 1.10–1.78, *p* = 0.007), indicating that higher ELAPSS scores, which reflect aneurysm growth risk, were strongly associated with alignment to neurovascular board decisions. For ChatGPT-3.5, the PHASES score showed a significant negative association (OR = 0.73, 95% CI: 0.55–0.97, *p* = 0.029), while severe comorbidities significantly reduced the likelihood of treatment recommendations in both models (OR = 0.047, *p* = 0.014 for ChatGPT-4 and OR = 0.002, *p* < 0.001 for ChatGPT-3.5). In contrast, no significant predictors were identified for Claude, and the analysis for Atlas GPT revealed unstable results with wide confidence intervals, suggesting lower robustness in these models.

## 4. Discussion

This study evaluates the performance of multiple AI models, including ChatGPT-4, ChatGPT-3.5, Claude, and Atlas GPT, in predicting treatment strategies for UIAs. Expert consensus and guideline recommendations underline the importance of multidisciplinary decision-making in the management of unruptured intracranial aneurysms. In daily practice, this is usually achieved through discussion in a dedicated neurovascular board, where neurosurgeons, neurointerventionalists, neuroradiologists, and neurologists contribute their perspectives. These boards combine established risk scores such as PHASES, ELAPSS, or UIATS with clinical judgement and patient-specific factors, allowing a balanced decision between rupture risk and treatment risk. On this basis, the neurovascular board is widely regarded as the reference standard for therapeutic recommendations in patients with UIA [7,18]. Our findings demonstrate that AI models might align with expert neurovascular board decisions, particularly in terms of overall treatment recommendations. This supports the growing evidence that AI, and specifically LLMs, can serve as valuable tools to assist in complex clinical decision-making processes and provide a first-line assessment and triage.

ChatGPT-4 achieved the highest overall accuracy, both in predicting conservative or operative therapy and in identifying the specific type of operative treatment. While ChatGPT-3.5 showed a stronger preference for clipping, and Claude and Atlas GPT leaned toward coiling, these variations reflect subtle differences in model outputs rather than significant deviations from clinical standards. Notably, the AI models demonstrated neither a conservative nor an aggressive tendency compared to the neurovascular board, reinforcing their ability to replicate expert-level decision-making.

AI models consistently recommended shorter follow-up intervals than the neurovascular board, which could have significant implications. While earlier imaging could help detect aneurysm growth or morphological change sooner and potentially lower rupture risk, it also has clear downsides. More frequent scans increase resource use and costs, place additional demands on radiology services, and, when CTA is the primary modality, add cumulative exposure to radiation and iodinated contrast [20,21]. For patients, this may translate into more outpatient clinic visits, interruptions to daily life, anxiety, and an increased chance of false positives leading to unnecessary investigations. Current guidelines emphasize individualized surveillance rather than fixed intervals, considering aneurysm and patient-specific risk [4,7]. Against this background, our findings indicate that although AI models generally agree with experts on whether to treat or observe, their tendency to suggest earlier re-imaging could shift practice toward over-surveillance if applied without modification. Integrating AI outputs with multidisciplinary board review and guideline-based protocols remains essential to balance vigilance with patient safety and efficient resource use.

The role of predictive risk scores, particularly the ELAPSS and PHASES scores, in guiding AI model outputs was also notable in our analysis. This observation reflects the importance of structured, evidence-based tools in enhancing AI performance, a finding that has been similarly reported in other AI-driven studies [15]. By integrating established clinical scoring systems, AI models can provide recommendations that are both interpretable and aligned with current standards of care.

Radiomics, the extraction of large amounts of quantitative features from medical imaging, has shown promise in characterizing aneurysm morphology, wall integrity, and other anatomical features that may correlate with rupture risk [22,23]. However, current AI-based language models are not yet capable of directly interpreting or analyzing raw imaging data. They therefore cannot independently generate radiomics-based predictions of UIA rupture risk. Our findings suggest that when provided with sufficiently detailed clinical and radiographic information, mirroring a radiomics-driven summary, AI models can offer recommendations that closely match expert decisions. This underscores the importance of integrating advanced imaging analytics with AI language models. This direction holds significant potential for improving the accuracy and reliability of rupture risk assessment in future clinical applications.

The accuracy observed in this study aligns with findings from other medical disciplines exploring AI applications. For example, recent studies have shown that GPT models can assist in neuro-oncology for diagnosing and determining treatment strategies for gliomas, with ChatGPT achieving a reasonable level of accuracy comparable to clinical experts [24]. Additionally, the use of AI in radiological interpretation and treatment planning has demonstrated its capability to support clinicians by reducing workload and enhancing efficiency [15]. Furthermore, it was shown that ChatGPT-3.5 responses on general neurosurgical topics were comparable to those of neurosurgeons with low seniority. In contrast, the assessment of ChatGPT 4.0 was comparable to that of neurosurgeons with high seniority [25]. These studies, coupled with our results, highlight the broad applicability of AI models across various neurosurgical and medical domains.

An important aspect to consider when interpreting AI model performance is inter-user variability. While AI models like ChatGPT can generate reliable outputs, the results are often influenced by the phrasing of prompts and the level of detail provided. In this study, we sought to minimize such variability by employing a rigorous, structured input design: each of the four LLMs received the same description of the expected response, as well as the same patient data and standardized prompts. This approach allowed us to generate more detailed and uniform responses across models. Nonetheless, it is worth noting that LLMs are continually evolving and adapting as their underlying databases expand, which introduces variability over time and presents an ongoing challenge to their reliability as clinical support tools. These considerations underscore the importance of clearly defined guidelines, consistent data presentation, and standardized prompting protocols when integrating AI into clinical workflows, particularly for complex cases like UIA management.

The findings of this study suggest that AI models, particularly ChatGPT-4, have the potential to serve as a valuable first-line triage tool to support clinical decision-making in the management of unruptured intracranial aneurysms. This is especially relevant in settings where healthcare providers may not frequently encounter UIAs or lack access to experienced neurovascular teams. By rapidly synthesizing clinical and radiographic data, AI can provide a preliminary recommendation that aligns closely with expert-level decisions. Such support could be beneficial in resource-limited settings, where access to specialized care is limited, or in emergencies that require swift preliminary assessments.

Nevertheless, it is crucial to acknowledge that AI models cannot supplant the expert judgment and collaborative decision-making processes offered by an interdisciplinary neurovascular board, which remains the cornerstone of UIA management. Neurovascular boards bring together diverse clinical perspectives, patient-specific considerations, and nuanced real-time discussions, factors that current AI models do not fully replicate. While AI can streamline preliminary evaluations by rapidly synthesizing large volumes of data, its role is best viewed as complementary to human expertise. Our results suggest that AI may serve as a first-line tool to help clinicians navigate complex cases. Still, it should not replace the in-depth deliberation and collective expertise characteristic of a multidisciplinary approach.

## 5. Limitations

Despite the encouraging results, it is essential to recognize several limitations in this study. First, each AI model analyzed is subject to ongoing updates and refinements, meaning that both inter-model variability and intra-model evolution are inevitable as their underlying datasets expand. This can lead to evolving and sometimes inconsistent outputs over time, even if the clinical scenario remains unchanged. Second, although we used a standardized format for data input, real-world clinical decision-making encompasses dynamic, multidisciplinary interactions and patient-specific factors that are not fully captured by retrospective data alone. Third, the recommendations produced by LLMs can vary significantly based on how prompts are formulated—slight differences in phrasing or the level of detail provided can substantially alter the generated responses. Fourth, our relatively small sample size may limit the generalizability of these findings, particularly for rarer treatment modalities such as flow diverters and WEB devices, which were each used in only two cases in our cohort. The low frequency of these modalities reflects their limited application in real-world practice at our center rather than selection bias; however, this small number inevitably restricts the precision of AI performance estimates in these subgroups, and results should therefore be interpreted with caution. Finally, exploring the performance of next-generation AI models, which may feature continuous learning capabilities, along with prospectively evaluating patient outcomes, will be crucial to fully understanding the actual impact of AI on clinical decision-making. Future studies incorporating larger, multi-center cohorts, real-time data, and patient-centered outcomes will help validate and refine the clinical utility of AI-driven decision support for the management of unruptured intracranial aneurysms.

## 6. Conclusions

In conclusion, our findings indicate that the evaluated LLMs, particularly ChatGPT-4, demonstrated high accuracy in determining whether conservative or operative management was appropriate for unruptured intracranial aneurysms. However, there was noticeably more variability when recommending specific treatment modalities (e.g., clipping vs. coiling). These results suggest that AI-based language models can be a valuable tool for initial screening decisions, especially in settings with limited access to specialized neurosurgical expertise. Nevertheless, the multidisciplinary neurovascular board, with its comprehensive and collaborative approach, remains essential for definitive management of unruptured intracranial aneurysms.

## Figures and Tables

**Table 1:** Descriptive Statistics of Patient Demographics, Aneurysm Characteristics, and Risk Scores.

Demographics	Range	Median (IQR)/Mean ± SD/n (%)
Age (years)	31–95	65.40 ± 12.51
Severe Comorbidities		15 (26.3%)
Aneurysm saccular Morphology		53 (93.0%)
Irregular Aneurysm		2 (4%)
Aneurysm location		
- Middle cerebral artery		14 (24.6%)
- Anterior cerebral artery		17 (29.8%)
- Internal carotid artery		24 (42.1%)
- Vertebral/posterior cerebral artery		2 (3.5%)
Intradural aneurysm location		48 (84.2%)
Growth under conservative treatment		4 (7.0%)
Aneurysm Size (mm)	2.0–25.0	6 (2.2–9.8)
Size Ratio	1.00–6.60	2.0 (0.5–3.5)
Aspect Ratio	0.66–8.50	3.0 (1.29–4.71)
PHASES Score	0–23	6.0 (1.0–11.0)
ELAPSS Score	2–29	14.63 ± 7.11
UIATS Favors Repair	2–15	8.0 (4.0–12.0)
Conservative treatment		26 (45.6%)
Treatment method		
- Operative clipping		14 (24.6%)
- Endovascular coiling		13 (22.8%)
- Endovascular flow diverter		2 (3.5%)
-Endovascular WEB device/coiling		2 (3.5%)

**Table 2:** Comparison of treatment decisions and follow-up recommendations between the neurovascular board and AI models. ChatGPT-3.5 favored clipping, whereas ChatGPT-4 demonstrated a more balanced approach. Claude and Atlas GPT leaned toward coiling and other endovascular treatments. AI models generally recommended shorter follow-up intervals for conservative management. All percentages are calculated using the entire study cohort (n = 57) as the denominator. Both absolute numbers (N) and percentages (%) are reported.

Model	Conservative (%)	WEB Device/Coiling (%)	Clipping (%)	Coiling (%)	Flow Diverter (%)	Mean Follow-Up in Months
Neurovascular Board	26 (45.6)	2 (3.5)	14 (24.6)	13 (41.9)	2 (3.5)	13.36
ChatGPT-4	24 (42.1)	3 (5.3)	13 (22.8)	17 (29.9)	0 (0.0)	10.00
ChatGPT-3.5	22 (38.6)	0 (0.0)	33 (57.9)	2 (3.5)	0 (0.0)	12.00
Claude	27 (42.1)	10 (17.5)	1 (1.8)	19 (33.3)	0 (0.0)	7.72
Atlas GPT	29 (50.9)	9 (15.78)	2 (3.5)	17 (29.9)	0 (0.0)	10.76

**Table 3:** (**A**) Left column: Accuracy of each AI model in correctly predicting conservative management for unruptured intracranial aneurysms. ChatGPT-4 achieved the highest accuracy at 89%, followed by ChatGPT-3.5, Atlas, and Claude. Right column: Pairwise comparisons of accuracy between AI models, with Bonferroni-adjusted *p*-values. ChatGPT -4 demonstrated significantly higher accuracy than Claude and Atlas, while the other comparisons were not statistically significant. (**B**) Left columns: Accuracy of each AI model in correctly predicting treatment type for unruptured intracranial aneurysms with ChatGPT-4 achieving the highest accuracy at 73%, followed by Atlas, Claude, and ChatGPT-3.5. Right columns: Pairwise comparisons of accuracy between AI models with Bonferroni-adjusted *p*-values. ChatGPT-4 demonstrated significantly higher accuracy than ChatGPT-3.5, while other comparisons were not statistically significant.

(A)					
AI Model	Mean Accuracy Conservative Prediction	Std. Deviation	Pair Comparison	*p*-Value	Significant with Bonferroni Adjustment?
ChatGPT-4	0.89	0.310	ChatGPT-4 vs. ChatGPT-3.5	0.160	No
ChatGPT-3.5	0.82	0.384	ChatGPT-4 vs. Claude GPT	< 0.001	Yes
Atlas GPT	0.74	0.444	Chatgpt-4 vs. Atlas GPT	0.002	Yes
Claude	0.70	0.462	GPT-3.5 vs. Claude	0.070	No
Cochran’s Q *p*-value	0.003		ChatGPT-3.5 vs. Atlas GPT	0.230	No
			Claude vs. Atlas GPT	0.310	No
(**B**)					
**AI Model**	**Mean Accuracy (Treatment Type)**	**Std. Deviation**	**Pair Comparison**	***p*-Value**	**Significant with Bonferroni Adjustment?**
ChatGPT-4	0.73	0.456	ChatGPT-4 vs. ChatGPT-3.5	0.002	Yes
ChatGPT-3.5	0.27	0.456	ChatGPT-4 vs. Claude	0.157	No
Claude	0.50	0.512	ChatGPT-4 vs. Atlas GPT	0.317	No
Atlas GPT	0.55	0.510	ChatGPT-3.5 vs. Claude	0.071	No
Cochran’s Q *p*-value	0.008		ChatGPT-3.5 vs. Atlas GPT	0.023	No
			Claude vs. Atlas GPT	0.564	No

## Data Availability

The data presented in this study are available on reasonable request from the corresponding author. The data are not publicly available due to ethical and privacy restrictions, as they contain clinical information derived from patient records.

## Supplementary Materials

The following supporting information can be downloaded at: https://www.mdpi.com/article/10.3390/brainsci15101061/s1, Supplementary data S1: Search engine data entry describing clinical and radiographic parameters entered into each Large Language Model (LLM), along with the corresponding outputs and the neurovascular board’s decisions.

## Abbreviations

ACA	Anterior cerebral artery
ACOM	Anterior communicating artery
AI	Artificial intelligence
ASA	American Society of Anesthesiologists physical-status score
CI	Confidence interval
DDR	Distal dural ring
ELAPSS	Aneurysm growth-risk score (Earlier SAH, Location, Age, Population, Size, Shape)
GPT	Generative Pre-Trained Transformer (e.g., GPT-4, GPT-3.5)
ICA	Internal carotid artery
LLM	Large language model
MCA	Middle cerebral artery
OR	Odds ratio
PHASES	Aneurysm rupture-risk score (Population, Hypertension, Age, Size, Earlier SAH, Site)
SAH	Subarachnoid haemorrhage
SD	Standard deviation
SPSS	Statistical Package for the Social Sciences
STROBE	Strengthening the Reporting of Observational Studies in Epidemiology
UIA	Unruptured intracranial aneurysm
UIATS	Unruptured Intracranial Aneurysm Treatment Score
WEB	Woven EndoBridge (intrasaccular flow-diversion device)
