# Supplementary material for: Comparative Analysis of AI Models in Predicting Treatment Strategies for Unruptured Intracranial Aneurysms
Source: Brain Sci. 2025 Sep 29;15(10):1061. doi: 10.3390/brainsci15101061 (PMC12563265; doi:10.3390/brainsci15101061)
Supplement: Supplementary file 1 [file brainsci-15-01061-s001.zip › brainsci-3857392-supplementary.pdf]

Supplementary data

Supplementary data S1:

Search engine data entry describing clinical and radiographic parameters entered into each Large Language Model (LLM), along with the corresponding outputs and the neurovascular board's decisions.

I am about to give you a series of clinical and radiographic data points for a patient with an unruptured intracranial aneurysm (UIA). Based on the information below, please respond by indicating whether you recommend conservative management (0) or operative treatment (1). If operative treatment is recommended (1), please specify which modality (0 = clipping, 1 = endovascular). If you recommend an endovascular approach (1), please choose one of the following: coiling (1), flow-diverter (2), or WEB device (3). If you recommend conservative management (0), please specify the follow-up interval (in months) until the next imaging/control.

Data:

- Age (years): [e.g., 65]
- Severe comorbidities (ASA Score ≥ 3) (0=no, 1=yes): [e.g., 1]
- Morphology (Fusiform=0, Saccular=1): [e.g., 1]
- Irregular aneurysm (no=0, yes=1): [e.g., 0]
- Location (intradural=1, extradural=0): [e.g., 1]
- Maximum aneurysm size (mm): [e.g., 8]
- Aneurysm location (1=MCA, 2=ACA, 3=ICA, 4=Vertebral, 5=Basilar): [e.g., 3]
- Multiple aneurysms (0=no, 1=yes): [e.g., 0]
- Size ratio (aneurysm size / average vessel diameter): [e.g., 2.5]
- Aspect ratio: [e.g., 2.0]
- Aneurysm growth documented (0=no, 1=yes): [e.g., 0]
- PHASES score: [e.g., 7]
- ELAPSS score: [e.g., 10]

Please provide your structured output in the following format:

1. Conservative (0) or operative (1)
2. If conservative: recommended follow-up interval (months)
3. If operative: clipping (0) or endovascular (1)
4. If endovascular: coiling (1), flow diverter (2), or WEB device (3)
